# Maternal smoking, consumption of alcohol, and caffeinated beverages during pregnancy and the risk of childhood brain tumors: a meta-analysis of observational studies

**DOI:** 10.1186/s12889-024-18569-9

**Published:** 2024-05-06

**Authors:** Zihao Hu, Jianbo Ye, Shenbao Shi, Chuangcai Luo, Tianwei Wang, Yang Liu, Jing’an Ye, Xinlin Sun, Yiquan Ke, Chongxian Hou

**Affiliations:** 1 The National Key Clinical Specialty, The Engineering Technology Research Center of Education Ministry of China, Guangdong Provincial Key Laboratory on Brain Function Repair and Regeneration, Department of Neurosurgery, Zhujiang Hospital, Southern Medical University, 510282 Guangzhou, People’s Republic of China; 2 Department of Neurosurgery, The Tenth Affiliated Hospital, Southern Medical University (Dongguan People’s Hospital), 523059 Dongguan, People’s Republic of China; 3 Department of Neurosurgery, Shenzhen People’s Hospital (The Second Clinical Medical College of Jinan University; The First Affiliated Hospital of Southern University of Science and Technology), 518020 Shenzhen, People’s Republic of China

**Keywords:** Maternal smoking, Maternal alcohol consumption, Maternal caffeinated beverages consumption, Childhood brain tumor (CBT), Glioma

## Abstract

**Background:**

We conducted this meta-analysis to investigate the potential association between maternal smoking, alcohol and caffeinated beverages consumption during pregnancy and the risk of childhood brain tumors (CBTs).

**Methods:**

A thorough search was carried out on PubMed, Embase, Web of Science, Cochrane Library, and China National Knowledge Internet to identify pertinent articles. Fixed or random effects model was applied to meta-analyze the data.

**Results:**

The results suggested a borderline statistically significant increased risk of CBTs associated with maternal smoking during pregnancy (OR 1.04, 95% CI 0.99–1.09). We found that passive smoking (OR 1.12, 95% CI 1.03–1.20), rather than active smoking (OR 1.00, 95% CI 0.93–1.07), led to an increased risk of CBTs. The results suggested a higher risk in 0–1 year old children (OR 1.21, 95% CI 0.94–1.56), followed by 0–4 years old children (OR 1.12, 95% CI 0.97–1.28) and 5–9 years old children (OR 1.11, 95% CI 0.95–1.29). This meta-analysis found no significant association between maternal alcohol consumption during pregnancy and CBTs risk (OR 1.00, 95% CI 0.80–1.24). An increased risk of CBTs was found to be associated with maternal consumption of caffeinated beverages (OR 1.16, 95% CI 1.07–1.26) during pregnancy, especially coffee (OR 1.18, 95% CI 1.00–1.38).

**Conclusions:**

Maternal passive smoking, consumption of caffeinated beverages during pregnancy should be considered as risk factors for CBTs, especially glioma. More prospective cohort studies are warranted to provide a higher level of evidence.

**Supplementary Information:**

The online version contains supplementary material available at 10.1186/s12889-024-18569-9.

## Introduction

There is limited understanding regarding the etiology of childhood brain tumors (CBTs), which are the most common solid tumors among children [1]. Evidence from animal studies has led to a hypothesis that the central nervous system is susceptible to carcinogenesis during the prenatal period [2]. Maternal exposures during pregnancy might play a crucial role in the risk of CBTs, as reported in two recent meta-analyses [3, 4].

In 2022, the prevalence of tobacco use among females aged 15 years and older was 7.4% [5]. A cumulative count of 83 constituents found in tobacco and tobacco smoke, including polycyclic aromatic hydrocarbons (PAHs) and tobacco-specific N-nitrosamines (TSNAs), have been categorized as carcinogens by the International Agency for Research on Cancer (IARC) [6]. The IARC has classified parental smoking as a causal factor for childhood leukaemia and childhood hepatoblastoma [7]. Furthermore, the presence of carcinogens in tobacco smoke might exert a more pronounced impact on fetuses and young children due to their underdeveloped blood-brain barrier [2]. Therefore, maternal smoking during pregnancy might be a potential cause of CBTs. Findings from prior studies investigating the association between maternal smoking during pregnancy and the risk of CBTs have shown inconclusive results [8–41]. In a meta-analysis published in 2014, no significant association was found between maternal smoking during pregnancy and risk of CBTs (odds ratio (OR) 0.96, 95% confidence interval (CI) 0.86–1.07) [42]. While, the latest meta-analysis reported that maternal smoking > 10 cigarettes per day during pregnancy (effect sizes 1.18, 95% CI 1.00–1.40) were associated with CBTs risk in cohort studies [4]. However, the four included cohort studies involve a duplicated population, leading to inaccurate results [43, 44]. In comparison to previous meta-analyses on this subject, the present study included more original studies with relatively high quality and avoided duplicated population. In addition, our current study also explored the correlation between maternal smoking during pregnancy and the risk of CBTs, while categorizing it by tumor category, quantity of cigarettes smoked, age at diagnosis, and the type of exposure (active/passive smoking).

In 2020, an estimated 4.1% of new cases of cancer worldwide were attributable to alcohol consumption [45]. Alcohol has been reported to be associated with various types of cancer, including liver cancer, colorectal cancer, and upper digestive tract tumors [46]. The exact mechanisms by which alcohol exerts carcinogenic effects are not fully understood. Possible mechanisms include the genotoxic effects of acetaldehyde, which can cause DNA damage [46, 47]. Alcohol can also cross the blood-brain barrier [48], which may be a risk factor for the central nervous system and warrant further investigation. Most studies suggest no significant association between maternal alcohol consumption and the risk of CBTs [12, 20, 29, 49–51]. While there are still some studies suggesting an increased risk, especially for beer consumption [11, 15, 52]. In this meta-analysis, we investigated the relationship between alcohol consumption during pregnancy and the risk of CBTs. Additionally, we conducted subgroup analyses based on the types of alcohol consumed and subtypes of brain tumors.

Coffee and tea are the most popular beverages worldwide. It has been reported that the consumption of coffee and tea is associated with various metabolic diseases, cardiovascular conditions, cancers, and so forth [53, 54]. Both coffee and tea contain caffeine [55]. The CARE Study Group has proved that caffeine is rapidly absorbed and readily passes the placental barrier [56]. Accumulating evidence from epidemiological studies showed that consumption of caffeine during pregnancy is associated with adverse gestational outcomes. In addition, caffeine exposure during pregnancy may induce epigenetic changes in the developing fetus [57]. Several studies have explored the association between maternal coffee and tea consumption during pregnancy and the risk of CBTs [29, 38, 50, 58–61]. However, the results are inconsistent. Evidence from the study conducted by Plichart et al. suggests that maternal consumption of coffee and tea during pregnancy might elevate the risk of CBTs [38]. Greenop et al. found that maternal consumption two or more cups of coffee a day during pregnancy is associated with an increased risk of CBTs [60]. On the other hand, Pogoda et al. reported no associations between brain tumor risk and maternal consumption of caffeine, but the results suggested a borderline increased risk tendency [61]. While three others found no significant associations with coffee, tea, or caffeinated beverages [29, 58, 59]. In the present study, we meta-analyzed these data to further explore such relationship.

The present study aimed to investigate the potential association between maternal smoking, alcohol and caffeinated beverages consumption during pregnancy and the risk of CBTs.

## Materials and methods

This meta-analysis follows the Preferred Reporting Items for Systematic Reviews and Meta-Analyses (PRISMA) statement [62].

### Literature search strategy

A thorough search was carried out on PubMed, Embase, Web of Science, Cochrane Library, and China National Knowledge Internet to identify pertinent articles published between January 1980 and February 2024. In the literature search for exposure of interest, we respectively employed the following search terms: (((maternal) OR (parental) OR (prenatal) OR (during pregnancy)) AND ((smoking) OR (cigarette) OR (tobacco))), (((maternal) OR (parental) OR (prenatal) OR (during pregnancy)) AND (alcohol)), (((maternal) OR (parental) OR (prenatal) OR (during pregnancy)) AND ((coffee) OR (caffeine) OR (tea))). In the literature search for outcome of interest, the following search terms were used: ((medulloblastoma) OR (craniopharyngioma) OR (ependymoma) OR (glioma) OR (glioblastoma) OR (meningioma) OR (acoustic neuroma) OR (pituitary adenoma) OR ((brain) OR (central nervous system) OR (childhood brain) OR (pediatric brain) OR (infant brain) OR (adolescent brain)) AND ((cancer) OR (tumor) OR (neoplasm))).

### Inclusion criteria and quality assessment

Following the PICOS principle, we applied the subsequent inclusion criteria: (1) The exposure of interest was maternal exposure to smoking, consumption of coffee, consumption of tea, and consumption of alcohol during pregnancy; (2) outcome of interest was CBTs; (3) case-control design or cohort design; (4) odds ratio (OR) or relative risk (RR) with 95% confidence intervals (CIs) was available; (5) written in English or Chinese. News, meta-analysis, and reviews were eliminated. Two investigators (ZH.H. and JB.Y.) retrieved the articles independently. Disagreements were resolved by a third investigator (CX.H.). The quality of the included studies was assessed using the Newcastle Ottawa Scale (NOS) [63]. Case-control studies with NOS scores less than 6 points and cohort studies with NOS scores less than 7 were excluded. Quality assessments were independently conducted by two researchers (ZH.H. and JB.Y.), and any disagreements were resolved by a third investigator (CX.H.).

### Data extraction

The following information was collected from the studies included in the present study: the last name of the first author, publication year, study design, study region, age and gender of participants, age at entry (cohort study), time of enrollment (cohort study), year of diagnosis, tumor category, number of cases and/or controls, OR or relative risk (RR) with corresponding 95% CI, data collection method, details of matching and adjustments made. For studies involving overlapping participants, we selectively extracted information. For instance, participants from the study conducted by Norman et al. [17] were encompassed within the study conducted by Filippini et al. [22]. The general impact of maternal smoking on the risk of CBTs was obtained from the study conducted by Filippini et al. [22], while the effects of different quantities of smoking on CBTs risk were extracted from the study by Norman et al. [17]. Data extraction was carried out independently by two investigators (ZH.H. and JB.Y.), and any discrepancies were resolved by a third investigator (CX.H.).

### Statistical analysis

Due to the relatively low incidence of brain tumors, the RR value exhibited a mathematical similarity to the OR value in the studies [64]. Therefore, for the sake of simplicity, the present study reported all effect sizes as OR values. We utilized either a fixed-effects model or a random-effects model to quantify the risk of brain tumors associated with maternal alcohol consumption, depending on the heterogeneity among studies [65]. Heterogeneity among the studies was assessed using the Q statistic and the I-squared (I^2^) value. The I^2^ value represents the portion of total variation attributed to differences among the studies rather than random error or chance. I^2^ values of 0%, 0–25%, 25–50%, and > 50% were categorized as indicating no, low, moderate, and high heterogeneity, respectively [66, 67]. Influence analysis was conducted to assess the significant influence of each study on the combined results by excluding each study one at a time. Publication bias was assessed using either Begg’s test (*n* ≥ 10) or Egger’s test (*n* < 10) depending on the number of involved studies [68]. Funnel plot was also conducted to evaluate the publication bias. All analyses were conducted using Stata 12.0 (StataCorp LLC, College Station, Texas, USA).

## Results

### Maternal smoking during pregnancy and risk of CBTs

#### Study selection and study characteristics

**Fig. 1:**
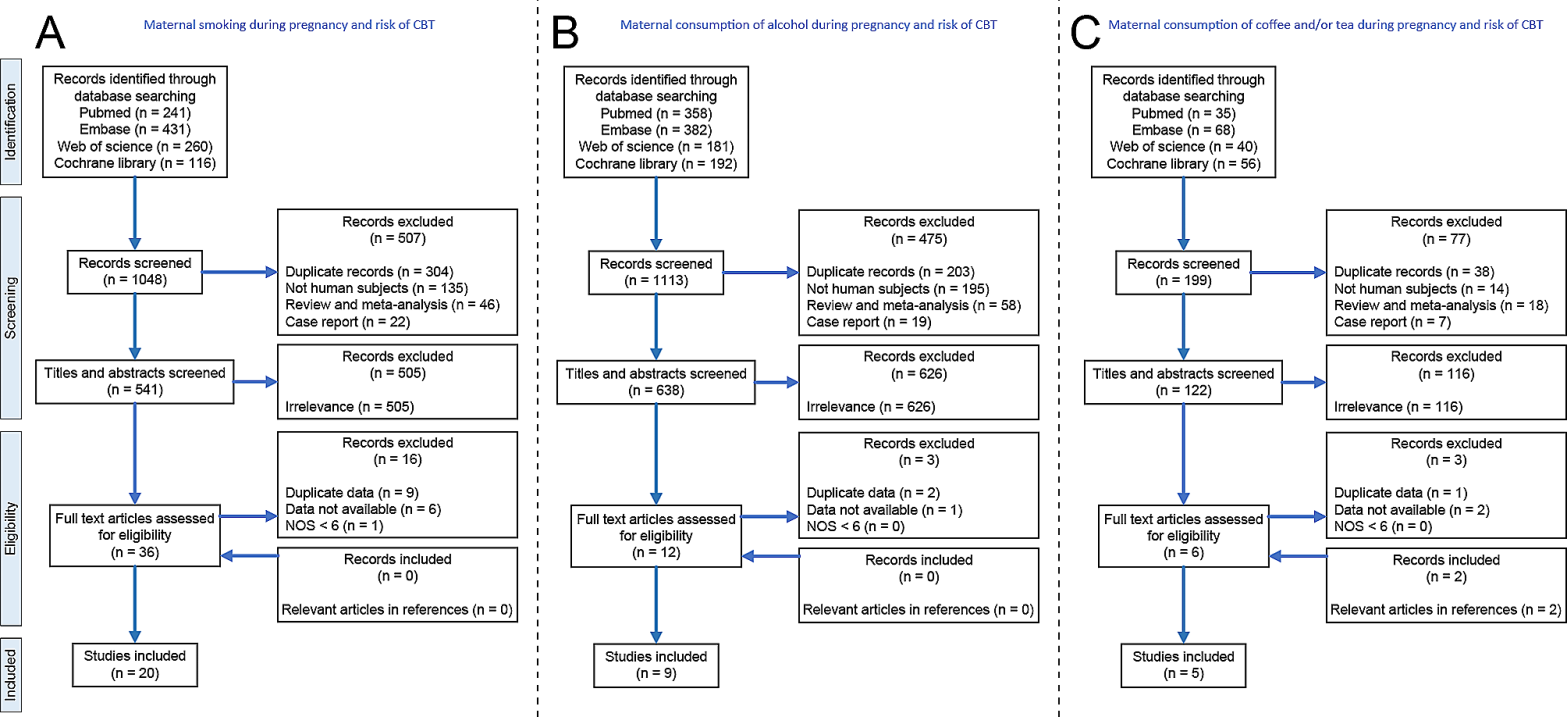
Flow diagram of study inclusion and exclusion. (**A**) Flow diagram of study assessment and selection on the association between maternal smoking during pregnancy and CBTs Risk. (**B**) Flow diagram of study assessment and selection on the association between maternal consumption of alcohol during pregnancy and CBTs Risk. (**C**) Flow diagram of study assessment and selection on the association between maternal consumption of caffeinated beverages during pregnancy and CBTs Risk. CBTs, childhood brain tumors

Following the retrieval strategy (Fig. 1A), this study includes 22 citations [8–29]. Among these, 20 are research articles [9, 11–29] with 17 case-control studies [8–23, 25, 28, 29] and 3 cohort studies [24, 26, 27], while 2 are comprised of comment-response pairs [8, 10]. The comments by McKinney et al. [8] and the response by Stjernfeldt et al. [10] provided supplementary data for the studies conducted by Sorahan et al. [21] and Stjernfeldt et al. [9]. The detailed characteristics of these studies are summarized in Table 1. The detailed NOS is shown in Supplementary Tables 1 and 2.

**Table 1:** Characteristics of included studies investigating the relationship between maternal smoking during pregnancy and the risk of CBTs

First author year	Study design	Country	Age (years)	Gender	Year of diagnosis	Tumor type	Adjustment or matched for	NOS
Stjernfeldt 1986 [9, 10]	case-control	Sweden	0–16	Both	1978–1981	CNS cancer	Adjusted for confounding factors (not mentioned).	6
McKinney 1986 [8, 21]	case-control	UK	< 15	Both	1980–1983	CNS tumor	Adjusted for other variables. Matched for age and gender.	6
Howe 1989 [11]	case-control	Canada	≤ 19	Both	1977–1983	CBTs	Adjusted for age at diagnosis. Matched for age and gender.	7
Kuijten 1990 [12]	case-control	USA	< 15	Both	1980–1986	Astrocytoma	Adjusted for demographic differences. Matched for age gender, race, and telephone exchange.	6
John 1991 [13]	case-control	USA	0–14	Both	1976–1983	Childhood cancer	Matched for age, gender, and geographic area.	7
Gold 1993 [14]	case-control	USA	< 18	Both	1977–1981	CBTs	Matched for age, gender, and mother’s racial/ethnic classification.	6
Bunin 1994 [15]	case-control	US and Canada	< 6	Both	1986–1989	Astrocytic glioma and PNET	Adjusted for income level. Matched for race, birth year, and telephone area code and prefix.	7
Hu 2000 [19]	case-control	China	≤ 18	Both	1991–1996	CBTs	Adjusted for mother’s education and family income. Matched for age, gender, and residence.	7
Filippini 2000 [18]	case-control	Italy	< 15	Both	1996–1997	CNS tumors	Adjusted for age, gender and residence. Matched for date of birth, gender, and residence area.	8
Schüz 2001 [20]	case-control	Germany	< 15	Both	1988–1993 1992–1994	CNS tumors	Adjusted for degree of urbanization and socioeconomic status. Matched for gender, date of birth, community.	7
Filippini 2002 [22]	case-control	9 centers*	0–19	Both	1976–1994	CBTs	Adjusted for age, gender, center. Matched for age, gender, and center.	7
Pang 2003 [23]	case-control	UK	< 15	Both	1991–1994	CNS tumors	Adjusted for parental age and deprivation. Matched for date of birth, gender, geographical area.	6
Milne 2012 [25]	case-control	Australia	0–14	Both	2005–2010	CBTs	Adjusted for matching variables, child’s ethnicity, year of birth group, mother’s age group, alcohol consumption during pregnancy, household income. Matched for age, gender and state of residence.	7
Vienneau 2016 [28]	case-control	4 countires^#^	7–19	Both	2004–2008	CBTs	Adjusted for maternal age and parental education. Matched for gender, age-group, geographical region.	7
Bailey 2017 [29]	case-control	France	< 15	Both	2003–2004 2010–2011	CBTs	Adjusted for matching factors and study of origin. Matched for age and gender.	6
Stavrou 2009 [24]	cohort study	Australia	0–12	Both	1994–2005^a^	CNS tumors	Adjusted for: Maternal smoking, Baby sex, Maternal age, Child’s age at diagnosis, Birth weight, Gestational age, ARIA?, IRSD, Maternal diabetes, Maternal hypertension, Gestational diabetes, Preeclampsia	8
Tettamanti 2016 [27]	cohort study	Sweden	< 15	Both	1983–2010^a^	CBTs	Adjusted for child’s sex, birth year, maternal age, maternal birthplace, and maternal educational level.	7
Heck 2016 [26]	cohort study	USA	≤ 5	Both	2007–2011^a^	Glioma	Adjusted for birth year, maternal race/ethnicity, and maternal years of education. Matched by year of birth.	8

CBTs, childhood brain tumors; CNS, central nervous system; PNET, primitive neuroectodermal tumor; UK, the United Kingdom; USA, The United States of America; * 9 centers: Paris, Milan, Valencia, Israel, Manitoba, Los Angeles, San Francisco, Seattle, New South Wales; # 4 countries: Denmark, Sweden, Norway and Switzerland; a, born between

#### Overall effect of maternal smoking during pregnancy on the risk of CBTs

**Fig. 2:**
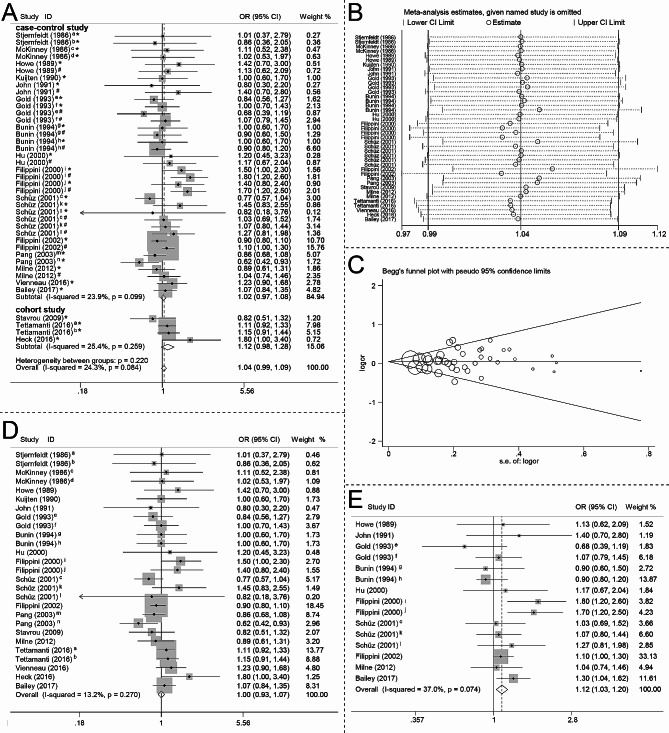
Forest plot, influence analysis and Begg’s funnel plot. (**A**) Forest plot of the association between maternal smoking during pregnancy and risk of CBTs. (**B**) Influence analysis on the meta-analyzed results by omitting each study. (**C**) Begg’s funnel plot. (**D**) Forest plot of the association between maternal active smoking during pregnancy and risk of CBTs. (**E**) Forest plot of the association between maternal passive smoking during pregnancy and risk of CBTs. CBTs, childhood brain tumors; CI, confidence interval; OR, odds ratio; a, 1–9 cigarettes/day; b, ≥ 10 cigarettes/day; c, 1–10 cigarettes/day; d, ≥ 11 cigarettes/day; e, < 1 pack/day; f, ≥ 1 pack/day; g, astrocytoma; h, primitive neuroectodermal tumor; i, conception period: the weeks before the mother learned she was pregnant during pregnancy; j, the period comprising the weeks after the mother knew she was pregnant; k, 11–20 cigarettes/day; l, > 20 cigarettes/day; m, 1–19 cigarettes/day; n, ≥ 20 cigarettes/day; *, exposed to maternal smoking; #, exposed to paternal smoking or maternal passive smoking

The meta-analyzed results suggested that maternal smoking during pregnancy was associated with a 4% increased risk of CBTs, although this difference did not reach statistical significance (OR 1.04, 95% CI 0.99–1.09, I^2^ 24.3%) (Fig. 2A). In addition, similar trends were seen in both case-control studies (OR 1.02, 95% CI 0.97–1.08, I^2^ 23.9%) and cohort studies (OR 1.12, 95% CI 0.98–1.28, I^2^ 25.4%) (Fig. 2A). Figure 2B illustrates the findings from the influence analysis. Begg’s test did not identify any significant publication bias (p = 0.84), and the corresponding funnel plot is presented in Fig. 2C.

#### Subgroup analysis of the association between maternal smoking during pregnancy and risk of CBTs

**Fig. 3:**
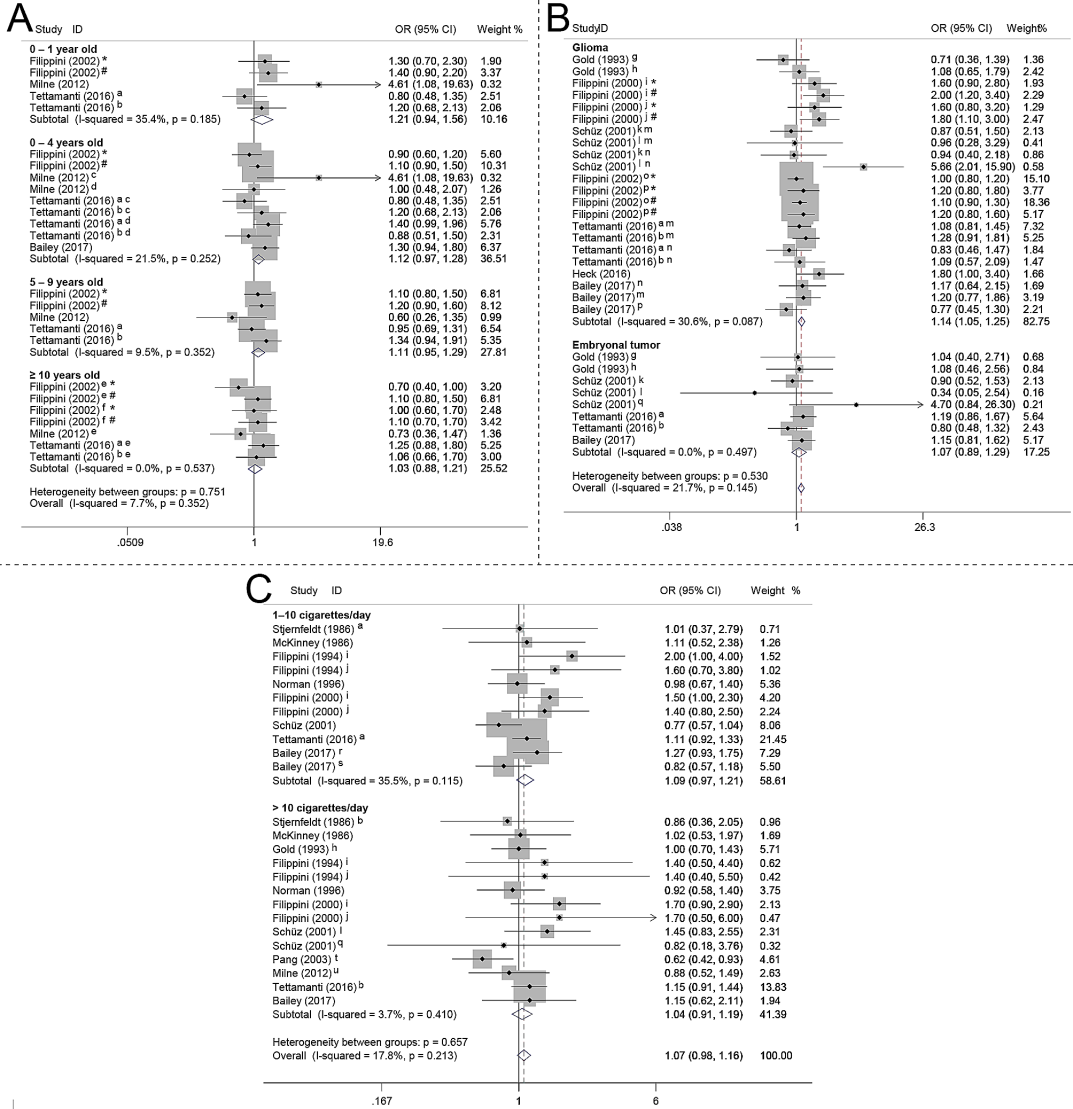
Forest plot of the subgroup analysis of the association between maternal smoking during pregnancy and risk of CBTs. (**A**) Forest plot of the association between maternal smoking during pregnancy and risk of CBTs stratified by age at diagnosis. (**B**) Forest plot of the association between maternal smoking during pregnancy and risk of CBTs stratified by tumor category. (**C**) Forest plot of the association between maternal smoking during pregnancy and risk of CBTs stratified by quantity of cigarettes smoked. CBTs, childhood brain tumors; CI, confidence interval; OR, odds ratio; a, 1–9 cigarettes/day; b, ≥ 10 cigarettes/day; c, 0–1 years old; d, 2–4 years old; e, 10–14 years old; f, 15–19 years old; g, < 1 pack/day; h, ≥ 1 pack/day; i, conception period: the weeks before the mother learned she was pregnant during pregnancy; j, the period comprising the weeks after the mother knew she was pregnant; k, 1–10 cigarettes/day; l, 11–20 cigarettes/day; m, astrocytoma; n, ependymoma; o, astroglial tumor; p, other glial tumor; q, > 20 cigarettes/day; r, < 5 cigarettes/day; s, 5–10 cigarettes/day; t, ≥ 20 cigarettes/day; u, ≥ 15 cigarettes/day; *, exposed to maternal smoking; #, exposed to paternal smoking or maternal passive smoking

No significant association was found between maternal active smoking during pregnancy and the risk of CBTs (OR 1.00, 95% CI 0.93–1.07, I^2^ 13.2%) (Fig. 2D). However, an increased risk of CBTs (OR 1.12, 95% CI 1.03–1.20, I^2^ 37.0%) (Fig. 2E) was observed with maternal passive smoking during pregnancy. In addition, from the presented data (Fig. 3A), we observed a consistent trend indicating an association between maternal smoking during pregnancy and CBTs risk stratified by age at diagnosis. Specifically, a trend was noticed showing an elevated risk of CBTs in younger age groups exposed to maternal smoking during pregnancy. The results suggested a higher risk in 0–1 year old children (OR 1.21, 95% CI 0.94–1.56, I^2^ 35.4%) (Fig. 3A), followed by 0–4 years old children (OR 1.12, 95% CI 0.97–1.28, I^2^ 21.5%) (Figs. 3) and 5–9 years old children (OR 1.11, 95% CI 0.95–1.29, I^2^ 9.5%) (Fig. 3A), albeit these associations did not reach statistical significance. Notably, no observable association was found between maternal smoking during pregnancy and the occurrence of CBTs among children older than 10 years (OR 1.03, 95% CI 0.88–1.21, I^2^ 0.0) (Fig. 3A). Therefore, the trend indicates a potential correlation where younger age at exposure to maternal smoking during pregnancy may correspond to an increased likelihood of CBTs risk.

We also investigated the association between maternal smoking during pregnancy and the risk of CBTs, stratified by tumor category (Fig. 3B) and the number of cigarettes smoked (Fig. 3C). The results suggested that maternal smoking during pregnancy is associated with increased risk of glioma (OR 1.14, 95% CI 1.05–1.25, I^2^ 30.6) (Fig. 3B). While no significant association was found between maternal smoking during pregnancy and risk of embryonal tumors (OR 1.07, 95% CI 0.89–1.29, I^2^ 0.0) (Fig. 3B). Moreover, the ORs for the association between CBTs risk and maternal smoking during pregnancy were 1.09 (95% CI, 0.97–1.21, I^2^ 35.5%) (Fig. 3C) for 1–10 cigarettes per day and 1.04 (95% CI, 0.91–1.19, I^2^ 3.7%) (Fig. 3C) for > 10 cigarettes per day, respectively.

### Maternal consumption of alcohol during pregnancy and the risk of CBTs

**Fig. 4:**
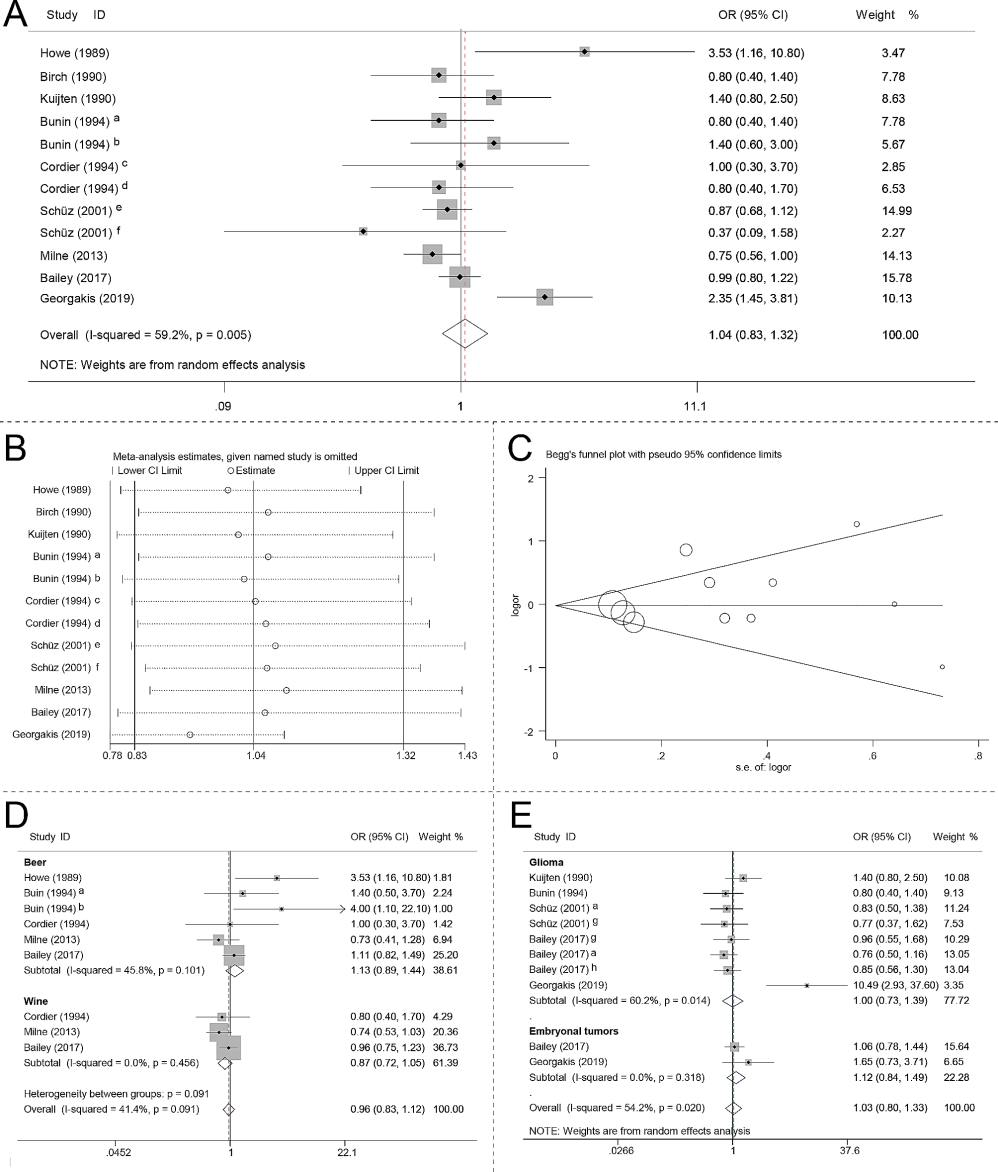
Forest plot, influence analysis and Begg’s funnel plot. (**A**) Forest plot of the association between maternal consumption of alcohol during pregnancy and risk of CBTs. (**B**) Influence analysis on the meta-analyzed results by omitting each study. (**C**) Begg’s funnel plot. (**B**) Forest plot of the association between maternal consumption of beer/wine during pregnancy and risk of CBTs. (**D**) Forest plot of the association between maternal consumption of alcohol during pregnancy and risk of CBTs stratified by tumor category. CBTs, childhood brain tumors; CI, confidence interval; OR, odds ratio; a, astrocytoma; b, primitive neuroectodermal tumor; c, consumption of beer; d, consumption of wine; e, 1–7 glasses/week; f, > 7 glasses/week; g, ependymoma; h, other glioma

Following the retrieval strategy (Fig. 1B), 8 case-control studies were involved. The detailed characteristics of these studies are summarized in Table 2. The detailed NOS is shown in Supplementary Table 3. Overall, this meta-analysis found no significant association between maternal alcohol consumption during pregnancy and CBTs risk (OR 1.00, 95% CI 0.80–1.24, I^2^ 54.1) (Fig. 4A). Figure 4B presents the results of the influence analysis. Egger’s test did not reveal any significant publication bias (p = 0.442), and the corresponding funnel plot is depicted in Fig. 4C.

**Table 2:** Characteristics of included studies investigating the relationship between maternal alcohol consumption during pregnancy and the risk of CBTs

First author year	Study design	Country	Age (years)	Gender	Year of diagnosis	Tumor type	Adjustment or matched for	NOS
Howe 1989 [11]	case-control	Canada	≤ 19	Both	1977–1983	CBTs	Adjusted for age at diagnosis. Matched for age and gender.	7
Birch 1990 [49]	case-control	UK	< 15	Both	1980–1983	CBTs	Matched for age and gender.	6
Kuijten 1990 [12]	case-control	USA	< 15	Both	1980–1986	Astrocytoma	Adjusted for demographic differences. Matched for age, race, and telephone area code and exchange.	7
Cordier 1994 [50]	case-control	France	< 15	Both	1985–1987	CBTs	Adjusted for child’s age and gender, maternal age, number of years of schooling of the mother. Matched for year of birth.	7
Bunin 1994 [15]	case-control	USA and Canada	< 6	Both	1986–1989	Astrocytoma and PNET	Adjusted for income level. Matched for race, birth year, and telephone area code and prefix.	7
Schüz 2001 [20]	case-control	Germany	< 15	Both	1988–1993 1992–1994	CBTs	Adjusted for degree of urbanization and socioeconomic status. Matched for gender, date of birth within 1 year, and community.	7
Milne 2013 [51]	case-control	Australia	0–14	Both	2005–2010	CBTs	Adjusted for matching variables, year of birth group, maternal age group, ethnicity, household income, maternal smoking. Matched for age, gender and state of residence.	7
Bailey 2017 [29]	case-control	France	< 15	Both	2003–2004 2010–2011	CBTs	Adjusted for age, gender and study of origin. Matched for age and gender.	7
Georgakis 2019 [52]	case-control	Greece	0–14	Both	2010–2016	CBTs	Adjusted for age, gender, maternal education, and a number of other factors. Matched for age, gender, and center.	6

CBTs, childhood brain tumors; NOS, Newcastle Ottawa Scale; PNET, primitive neuroectodermal tumor; USA, the United States of America. PNET, Primitive neuroectodermal tumor

The ORs for the association between CBTs risk and maternal consumption of alcohol during pregnancy were 0.87 (95% CI 0.72–1.05, I^2^ 0.0) (Fig. 4D) for wine consumption and 1.07 (95% CI 0.84–1.37, I^2^ 20.8) (Fig. 4D) for beer consumption. In subgroup analysis stratified by tumor category, no significant association was found between maternal consumption of alcohol and risk of glioma (OR 1.00, 95% CI 0.73–1.39, I^2^ 60.2) (Fig. 4E). In addition, a 12% higher risk of embryonal (OR 1.12, 95% CI 0.84–1.49, I^2^ 0.0) (Fig. 4E), even though not statistically significant, was found for maternal consumption of alcohol during pregnancy.

### Maternal consumption of coffee and/or tea during pregnancy and CBTs risk

**Fig. 5:**
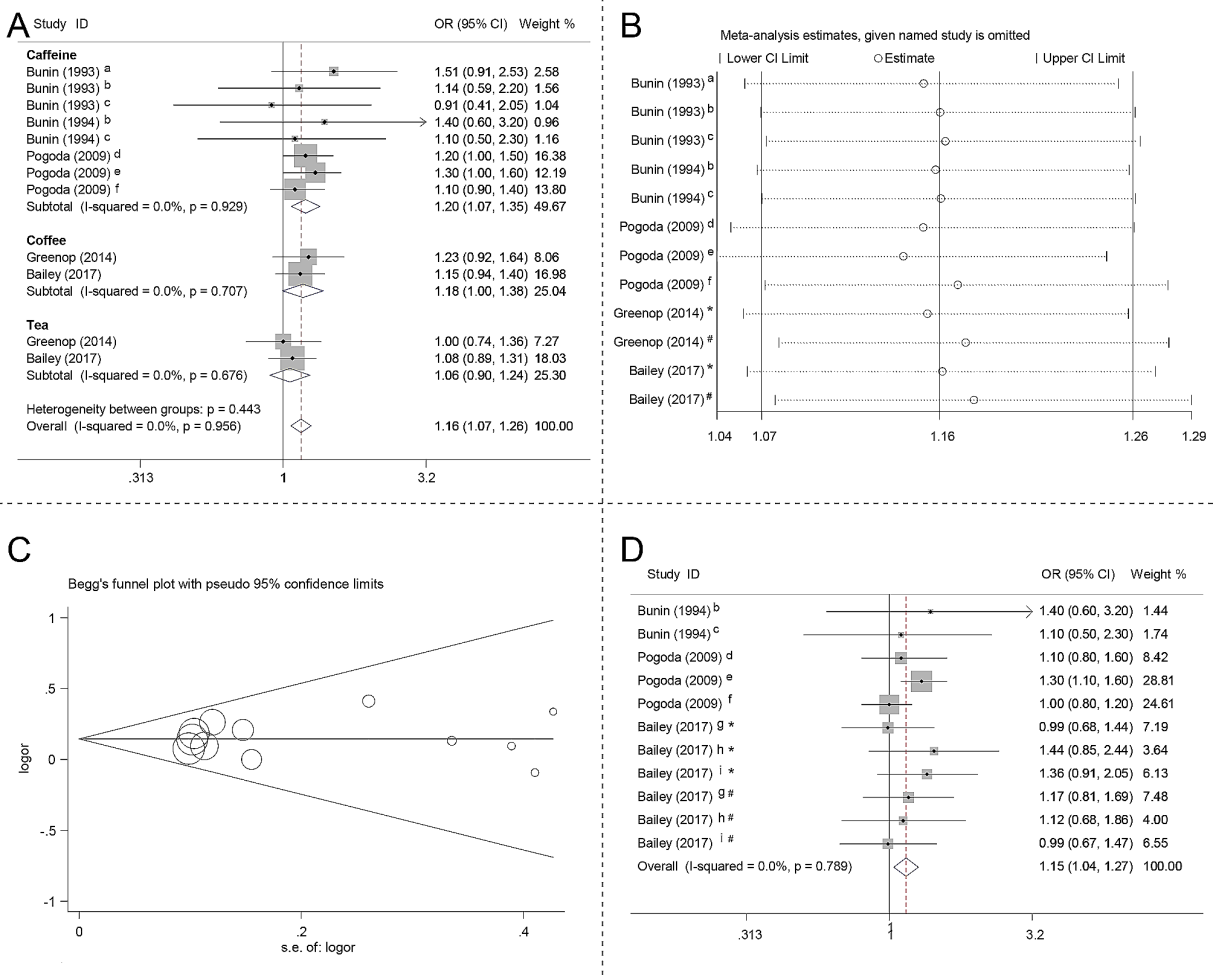
Forest plot, influence analysis and Begg’s funnel plot. (**A**) Forest plot of the association between maternal consumption of caffeinated beverages during pregnancy and risk of CBTs. (**B**) Influence analysis on the meta-analyzed results by omitting each study. (**C**) Begg’s funnel plot. (**D**) Forest plot of the association between maternal consumption of caffeinated beverages during pregnancy and risk of childhood glioma. CBTs, childhood brain tumors; CI, confidence interval; OR, odds ratio; a, 1 to 7 coffee equivalents per week; b, 8 to 14 coffee equivalents per week; c, > 14 coffee equivalents per week; d, 2nd exposure level; e, 3rd exposure level; f, 4th exposure level; g, astrocytoma; h, ependymoma; i, other glioma; *, coffee consumption; # tea consumption

Based on the retrieval strategy (Fig. 1C), a total of 5 case-control studies were included. The detailed characteristics of the involved studies are summarized in Table 3. The detailed NOS is shown in Supplementary Table 4. In our meta-analysis, increased risk of CBTs was found to be associated with maternal consumption of caffeinated beverages (OR 1.16, 95% CI 1.07–1.26, I^2^ 0.0) (Fig. 5A). In addition, maternal consumption of coffee (OR 1.18, 95% CI 1.00–1.38, I^2^ 0.0) during pregnancy was associated with an increased risk of CBTs. While, no significant association was found between maternal consumption of tea and risk of CBTs (OR 1.06, 95% CI 0.90–1.24, I^2^ 0.0) (Fig. 5A). Figure 5B presents the results of the influence analysis. Egger’s test did not reveal any significant publication bias (p = 0.743), and the corresponding funnel plot is depicted in Fig. 5C. In subgroup analysis, we found that increased risk of glioma is associated with maternal consumption of caffeinated beverages during pregnancy (OR 1.15, 95% CI 1.04–1.27, I^2^ 0.0) (Fig. 5D). The summary of the results in this study is shown in Table 4.

**Table 3:** Characteristics of included studies investigating the relationship between maternal consumption of caffeinated beverages during pregnancy and the risk of CBTs

First author year	Study design	Country	Age (years)	Gender	Year of diagnosis	Tumor type	Adjustment or matched for	NOS
Bunin 1993 [59]	case-control	USA and Canada	< 6	Both	1986–1989	PNET	Adjusted for income level. Matched for telephone area code and telephone number, date of birth, and race.	7
Bunin 1994 [58]	case-control	USA and Canada	< 6	Both	1986–1989	Astrocytoma	Adjusted for income level. Matched for telephone area code and telephone number, date of birth, and race.	6
Pogoda 2009 [61]	case-control	7 countries*	0–19	Both	1976–1992	CBTs	Adjusted for other exposure variables. Matched for region of residence, age, and gender.	7
Greenop 2014 [60]	case-control	Australia	< 15	Both	2005–2010	CBTs	Adjusted for child’s age, gender, state of residence, year of birth group, ethnicity, maternal age group, best education of either parent, maternal alcohol consumption during pregnancy. Matched for age, gender and state of residence.	8
Bailey 2017 [29]	case-control	France	< 15	Both	2003–2004 2010–2011	CBTs	Adjusted for age, gender and study of origin. Matched for age and gender.	7

CBTs, childhood brain tumors; PNET, PNET, primitive neuroectodermal tumor; USA, The United States of America. * Seven countries: USA, Israel, Italy, Spain, Australia, France, and Canada

**Table 4:** Summary of the results of this study

	OR (95% CI)	I^2^	Begg (P value)	Egger (P value)
**Maternal smoking during pregnancy**	1.04 (0.99–1.09)	24.3%	0.840	0.450
Study design				
Case-control studies	1.02 (0.97–1.08)	23.9%	0.794	0.402
Cohort studies	1.12 (0.98–1.28)	25.4%	0.734	0.819
Type of exposure				
Active smoking	1.00 (0.93–1.07)	13.2%	0.441	0.468
Passive smoking	1.12 (1.03–1.20)	37.0%	0.843	0.629
Age at diagnosis				
0–1 year old	1.21 (0.94–1.56)	35.4%	0.462	0.231
0–4 years old	1.12 (0.97–1.28)	21.5%	0.602	0.657
5–9 years old	1.11 (0.95–1.29)	9.5%	0.462	0.234
≥10 years old	1.03 (0.88–1.21)	0.0%	0.072	0.140
Tumor category				
Glioma	1.14 (1.05–1.25)	30.6%	0.652	0.155
Embryonal tumors	1.07 (0.89–1.29)	0.0%	0.711	0.875
Quantity of cigarettes smoked				
1–10 cigarette(s)/day	1.09 (0.97–1.21)	35.5%	0.436	0.349
>10 cigarettes/day	1.04 (0.91–1.19)	3.7%	0.661	0.659
**Maternal consumption of alcohol during pregnancy**	1.04 (0.83–1.32)	59.2%	0.891	0.442
Type of alcohol				
Beer	1.13 (0.89–1.44)	45.8%	0.260	0.274
Wine	0.87 (0.72–1.05)	0.0%	1.000	0.669
Tumor category				
Glioma	1.00 (0.73–1.39)	60.2%	0.386	0.029
Embryonal tumors	1.12 (0.84–1.49)	0.0%	1.000	N/A
**Maternal consumption of caffeinated beverages during pregnancy**	1.16 (1.07–1.26)	0.0%	0.732	0.743
Type of exposure				
Caffeine	1.20 (1.07–1.35)	0.0%	0.902	0.960
Coffee	1.18 (1.00–1.38)	0.0%	1.000	N/A
Tea	1.06 (0.90–1.24)	0.0%	1.000	N/A
Tumor category				
Glioma	1.15 (1.04–1.27)	0.0%	0.436	0.812

Begg, Begg’s test; CI, confidence interval; Egger, Egger’s test; N/A, Not Applicable; OR, odds ratio

## Discussion

Smoking, alcohol consumption, and consumption of caffeinated beverages have become common lifestyles for people. In recent decades, studies have explored the relationship between maternal exposure to these factors during pregnancy and the risk of childhood brain tumors, the most common solid tumor in children. This study aimed to compile data to provide clues and evidence for the prevention of childhood brain tumors.

### Maternal smoking during pregnancy and the risk of CBTs

Findings from prior studies investigating the association between maternal smoking during pregnancy and the risk of CBTs have shown inconclusive results. The results of the current meta-analysis indicated a borderline statistically significant increased risk of CBTs associated with maternal smoking during pregnancy (OR 1.04, 95% CI 0.99–1.09), which is inconsistent with previous meta-analyses [4, 42] and the results from the conference in 2022 [69]. Furthermore, the meta-analyzed results of cohort studies also showed increased risk of CBTs (OR 1.12, 95% CI 0.98–1.28). However, the three prospective studies which largely avoided recall bias all lacked data on potential confounding factors that could impact the risk of CBTs [24, 26, 27]. Findings derived from the large Swedish cohort study indicate that while maternal smoking during pregnancy has a limited overall effect on risk of CBTs, it may increase the risk of astrocytomas [27]. When we conducted subgroup analyses for active and passive smoking during pregnancy separately, we found that passive smoking (OR 1.12, 95% CI 1.03–1.20), rather than active smoking (OR 1.00, 95% CI 0.93–1.07), led to an increased risk of CBTs. Some studies demonstrated that passive smoking, but not active smoking, is associated with increased risk of some cancers [70, 71]. While, some studies reported that both active smoking and passive smoking increased cancer risk [72, 73]. However, these findings do not imply encouragement for active smoking during pregnancy. Such results may be influenced by confounding factors, although it cannot be ruled out that women might have a higher tolerance for active smoking.

In this meta-analysis, for studies that did not explicitly specify passive smoking, maternal exposure to paternal smoking during pregnancy was defined as passive smoking. Furthermore, a statistically significant association was identified in cases of glioma (OR 1.14, 95% CI 1.05–1.25). Additionally, in this study, no dose-response relationship was found between the number of cigarettes smoked by mothers during pregnancy and the risk of brain tumor incidence. These results suggest that during pregnancy, reducing the amount or frequency of smoking may not decrease the risk of childhood brain tumors. Instead, quitting smoking is necessary. In the present study, we also noticed a consistent pattern suggesting a link between maternal smoking during pregnancy and the risk of CBTs, particularly in younger age groups at the time of diagnosis. In addition, mothers who smoked during pregnancy are more likely to smoke after delivery. Therefore, it can also be further speculated that maternal smoking during pregnancy may have a greater impact on the child than after delivery.

### Maternal consumption of alcohol during pregnancy and risk of CBTs

Our meta-analysis did not find any statistically significant association between maternal alcohol consumption during pregnancy and the incidence of CBTs (OR 1.04, 95% CI 0.83–1.32). Interestingly, when we conducted subgroup analysis on different types of alcohol consumption, we observed a trend indicating a potential decreased risk of CBTs with wine consumption (OR 0.87, 95% CI 0.72–1.05), although this finding did not reach statistical significance. Unlike other alcoholic beverages, low-to-moderate wine consumption can reduce the incidence of cardiovascular diseases, type 2 diabetes, and lower the risk of certain tumors [74, 75]. However, there is still insufficient evidence at present to definitively classify consumption of wine as part of a healthy lifestyle. Howe et al. and Bunin et al. found that maternal beer consumption during pregnancy is associated with increased risk of CBTs [11, 15]. However, the results of the present meta-analysis suggested no statistically significant association (OR 1.13, 95% CI 0.89–1.44). Furthermore, neither glioma risk (OR 1.00, 95% CI 0.73–1.39) nor embryonal tumor risk (OR 1.12, 95% CI 0.84–1.49) was significantly associated with maternal consumption of alcohol during pregnancy.

While our meta-analysis suggests that there is no significant association between maternal alcohol consumption and the risk of CBTs, it is important to interpret these conclusions cautiously due to the fact that all the studies included in our analysis were case-control studies. Additionally, the number of studies included in this meta-analysis is small, highlighting the need for larger and less biased studies in the future to validate these findings. Specifically, prospective cohort studies would be valuable in providing more robust evidence regarding the potential link between maternal alcohol consumption during pregnancy and the risk of CBTs. Furthermore, it is important to note that while some current research results suggest that moderate alcohol consumption may reduce the risk of CBTs, it does not change the overall understanding of alcohol’s impact on public health. The World Health Organization still considers alcohol to increase the risk of cancer, regardless of the amount consumed [76, 77]. There is strong evidence linking alcohol consumption to an increased risk of breast, liver, oral, and colorectal cancer in adults [78, 79]. Therefore, it is still advisable to avoid alcohol consumption during pregnancy since it is related with cognitive defects and fetal alcohol spectrum disorders [80].

### Maternal caffeinated beverages consumption during pregnancy and risk of CBTs

Due to the limited number of studies investigating the relationship between maternal consumption of caffeinated beverages during pregnancy and the risk of CBTs, as well as the inclusion of studies utilizing overlapping population data that needed to be excluded [38, 50], only five case-control studies were involved in the present meta-analysis [29, 58–61]. Among these studies, two of them reported the intake of coffee and tea [29, 60]. As both coffee and tea contain caffeine, in these studies, coffee and tea were categorized as caffeinated beverages [55]. The remaining three studies classified caffeine as the exposure factor but did not specifically report the information of coffee and tea consumption [58, 59, 61]. Our results indicate that maternal caffeinated beverages consumption during pregnancy may increase the risk of CBTs (OR 1.16, 95% CI 1.07–1.26). Subgroup analysis of tumor category showed a similar trend in gliomas (OR 1.15, 95% CI 1.04–1.27), which is consistent with the conclusions of two previous meta-analyses on the relationship between coffee and tea intake and the risk of adult gliomas [81, 82]. No significant association was found between tea consumption during pregnancy and the risk of CBTs (OR 1.06, 95% CI 0.90–1.24). Differences in manufacturing processes and different types of coffee and tea may play different roles in the progression of cancer [83]. Individuals may also change their preference for coffee types, and different conclusions may be drawn due to regional differences in coffee preferences. However, currently, there is a lack of research on the risk of CBTs associated with maternal consumption of different types of coffee.

Until now, no explicit explanations have been given to explain the association between maternal caffeinated beverages consumption and increased risk of CBTs. Both coffee and tea contain caffeine. Caffeine and its related substances could inhibit DNA topoisomerase II (topo II), which plays an important role in cell growth and division [84]. Topo II inhibition may result in chromosomal aberrations and translocations, speculated to contribute to the pathogenesis of infant tumors. Ross et al. reported a positive association between maternal intake of Topo II inhibitors during pregnancy and the development of infant tumors [85]. On one hand, numerous studies suggest that caffeine consumption might act as a protective factor against various cancers [86–88]. On the other hand, several observational studies and most Mendelian Randomization studies did not provide sufficient evidence for a causal role of coffee or caffeine on these health outcomes [89–91].

### Bias, limitations and strengths

The following aspects might contribute to bias to the involved original studies: (1) Most of the involved studies were case-control studies which cannot avoid recall bias. It is difficult for parents to correctly remember their lifestyle 10 years (or more) before the studies. In addition, case mothers were more likely to over-report their exposure because they might be more inclined to consider smoking and consumption of beverages (alcohol, coffee, or tea) as a risk factors. (2) Mothers might under-report their exposure to smoking and beverages (alcohol, coffee, or tea) during pregnancy because they may not want to admit or be accused of harming the child. (3) About 20–50% of female smokers attempt to quit smoking during pregnancy, but half of them will fail. Women who fail to quit smoking typically go through a cycle of trying to decrease or quit, then relapsing, and making renewed attempts to quit. Therefore, in this situation, it is difficult for the studies to collect precise information about smoking [92, 93]. In addition, mothers who smoked during pregnancy are more likely to smoke also before conception and after delivery. However, the present study did not explore the association between maternal smoking before conception, after delivery and risk of CBTs. (4) Women classified as nonsmokers might have been exposed to passive smoking, potentially diminishing the effect of maternal smoking during pregnancy. (5) There is a possibility that children with CBTs, exposed to parental smoking, may be more active and may more frequently go to the hospital for physical examination, which might bring selection bias to the studies.

This study has some limitations: (1) The majority of the studies involved in the current meta-analysis were case-control studies, demonstrating an association rather than causality. (2) Some involved studies reported the data that could be used for subgroup analysis, while some other studies did not report such data. Thus, the results of subgroup analyses may not represent all the populations of the involved studies. (3) The number of studies regarding maternal alcohol and caffeinated beverages consumption, as well as the sample sizes in many subgroup analyses, is still insufficient. (4) Mothers exposed to maternal smoking and consumption of beverages during pregnancy are more likely to be exposed to these factors both before conception and after delivery. However, the current study did not investigate the correlation between exposure to these factors before conception, post-delivery, and the risk of CBTs. Therefore, these findings cannot precisely represent the exposure of mothers during pregnancy.

The strengths of this study include: (1) The present study is the largest meta-analysis to date that investigated the association between maternal smoking, alcohol, and caffeinated beverages consumption during pregnancy and risk of CBTs. In this study, we performed a comprehensive literature search. We reviewed the references of relevant literature to avoid any omissions. In addition, quality control was conducted on the literature. (2) This meta-analysis avoided the inclusion of duplicate populations when combining effect sizes. (3) We conducted multiple subgroup analyses to further investigate the relationship between exposure factors and the disease.

## Conclusions

In conclusion, the current meta-analysis revealed an association between passive smoking during pregnancy, rather than active smoking during pregnancy, and an increased risk of CBTs. Furthermore, maternal smoking during pregnancy is associated with an elevated risk of childhood glioma. In addition, a trend was noticed showing an elevated risk of CBTs in younger age groups exposed to maternal smoking during pregnancy. Moreover, maternal caffeinated beverages consumption is associated with an increased risk of CBTs, especially glioma. The results of the present meta-analysis suggest no significant association between maternal alcohol consumption and the risk of CBTs. Because of the limitations of the present study, more large well-designed prospective cohort studies and Mendelian Randomization studies with large sample size are warranted to provide a higher level of evidence.

### Electronic supplementary material

Below is the link to the electronic supplementary material.

Supplementary Material 1

Supplementary Material 2

## Data Availability

The datasets used during the current study are available from the corresponding author on reasonable request.
