# Supplementary material for: Maternal smoking, consumption of alcohol, and caffeinated beverages during pregnancy and the risk of childhood brain tumors: a meta-analysis of observational studies
Source: BMC Public Health. 2024 May 6;24:1238. doi: 10.1186/s12889-024-18569-9 (PMC11071245; doi:10.1186/s12889-024-18569-9)
Supplement: Supplementary file 2 — Supplementary Material 2 [file 12889_2024_18569_MOESM2_ESM.docx]

**Supplementary Table 1. Maternal smoking during pregnancy and the risk of childhood brain tumors (case-control studies).**

| Study | Selection |  |  |  | Comparability | Exposure |  |  | Total score |
| --- | --- | --- | --- | --- | --- | --- | --- | --- | --- |
|  | (1) Is the case definition adequate | (2) Representative of cases | (3) Selection of controls | (4) Definition of controls | (1) Comparability of cases and controls obtained based on the design or analysis | (1) Determination of exposure | (2) Whether the same ascertainment method was used for the exposure of the cases and controls | (3) No response rate |  |
| Stjernfeldt 1986 [9] | 0 | * | * | * | ** | 0 | * | 0 | 6 |
| McKinney 1986 [8] | * | * | * | * | * | 0 | * | 0 | 6 |
| Howe 1989 [11] | * | * | * | * | ** | 0 | * | 0 | 7 |
| Kuijten 1990 [12] | 0 | * | * | * | ** | 0 | * | 0 | 6 |
| John 1991 [13] | 0 | * | * | * | ** | 0 | * | * | 7 |
| Gold 1993 [14] | 0 | * | * | * | ** | 0 | * | 0 | 6 |
| Bunin 1994 [15] | 0 | * | * | * | ** | 0 | * | * | 7 |
| Hu 2000 [19] | 0 | * | * | * | ** | 0 | * | * | 7 |
| Filippini 2000 [18] | 0 | * | * | * | ** | * | * | * | 8 |
| Schüz 2001 [20] | * | * | * | * | ** | 0 | * | 0 | 7 |
| Filippini 2002 [22] | * | * | * | * | ** | 0 | * | 0 | 7 |
| Pang 2003 [23] | 0 | * | * | * | ** | 0 | * | 0 | 6 |
| Milne 2012 [25] | * | * | * | * | ** | 0 | * | 0 | 7 |
| Vienneau 2016 [28] | * | * | * | * | ** | 0 | * | 0 | 7 |
| Bailey 2017 [29] | 0 | * | * | * | ** | * | * | 0 | 7 |

Each study could receive at most one "*" for each entry on "Selection" and "Exposure". Items on "Comparability" can received up to two "*". The quality assessment values ranged from 0 to 9 scores.

**Supplementary Table 2. Maternal smoking during pregnancy and the risk of childhood brain tumors (cohort studies).**

| Study | Selection |  |  |  | Comparability | Outcome |  |  | Total score |
| --- | --- | --- | --- | --- | --- | --- | --- | --- | --- |
|  | (1) Representative of the exposure cohort | (2) Selection of the non-exposed cohort | (3) Determination of exposure | (4) No study subjects had outcome events prior to study entry | (1) Comparability of the obtained cohorts based on the design or analysis | (1) Assessment of the outcome events | (2) Is follow-up adequate for the occurrence of outcome | (3) Completeness of follow-up |  |
| Stavrou 2009 [24] | * | * | * | 0 | ** | * | * | * | 8 |
| Tettamanti 2016 [27] | * | * | * | 0 | ** | * | 0 | * | 7 |
| Heck 2016 [26] | * | * | * | 1 | ** | * | 0 | * | 8 |

Each study could receive at most one "*" for each entry on "Selection" and "Outcome". Items on "Comparability" can received up to two "*". The quality assessment values ranged from 0 to 9 scores.

**Supplementary Table 3. Maternal alcohol consumption during pregnancy and the risk of childhood brain tumors.**

| Study | Selection |  |  |  | Comparability | Exposure |  |  | Total score |
| --- | --- | --- | --- | --- | --- | --- | --- | --- | --- |
|  | (1) Is the case definition adequate | (2) Representative of cases | (3) Selection of controls | (4) Definition of controls | (1) Comparability of cases and controls obtained based on the design or analysis | (1) Determination of exposure | (2) Whether the same ascertainment method was used for the exposure of the cases and controls | (3) No response rate |  |
| Bailey 2017 [29] | 0 | * | * | * | ** | * | * | 0 | 7 |
| Georgakis 2019 [52] | 0 | * | * | * | ** | 0 | * | 0 | 6 |
| Kuijten 1990 [12] | * | * | * | * | ** | 0 | * | 0 | 7 |
| Milne 2013 [51] | * | * | * | * | ** | 0 | * | 0 | 7 |
| Schüz 2001 [20] | * | * | * | * | ** | 0 | * | 0 | 7 |
| Bunin 1994 [15] | * | * | * | * | * | 0 | * | * | 7 |
| Birch 1990 [49] | 0 | * | * | * | ** | 0 | * | 0 | 6 |
| Howe 1989 [11] | * | * | * | * | ** | 0 | * | 0 | 7 |
| Cordier 1994 [50] | * | * | * | * | ** | 0 | * | 0 | 7 |

Each study could receive at most one "*" for each entry on "Selection" and "Exposure". Items on "Comparability" can received up to two "*". The quality assessment values ranged from 0 to 9 scores.

**Supplementary Table 4. Maternal coffee and tea consumption during pregnancy and the risk of childhood brain tumors.**

| Study | Selection |  |  |  | Comparability | Exposure |  |  | Total score |
| --- | --- | --- | --- | --- | --- | --- | --- | --- | --- |
|  | (1) Is the case definition adequate | (2) Representative of cases | (3) Selection of controls | (4) Definition of controls | (1) Comparability of cases and controls obtained based on the design or analysis | (1) Determination of exposure | (2) Whether the same ascertainment method was used for the exposure of the cases and controls | (3) No response rate |  |
| Bunin1993 [59] | * | * | * | * | ** | 0 | * | 0 | 7 |
| Bunin1994 [58] | 0 | * | * | * | ** | 0 | * | 0 | 6 |
| Pogoda 2009 [61] | 0 | * | * | * | ** | * | * | 0 | 7 |
| Greenop 2014 [60] | * | * | * | * | ** | * | * | 0 | 8 |
| Bailey 2017 [29] | 0 | * | * | * | ** | * | * | 0 | 7 |

Each study could receive at most one "*" for each entry on "Selection" and "Exposure". Items on "Comparability" can received up to two "*". The quality assessment values ranged from 0 to 9 scores.
